# Preliminary study of a Digital Brazilian Portuguese Speech Assessment Instrument: validity and reliability of imitation and naming tasks

**DOI:** 10.1590/2317-1782/e20250084en

**Published:** 2026-01-30

**Authors:** Eduarda Pinheiro Oliveira, Dara da Silva Oliveira, Isabel da Rosa Alvaro, Letícia Bitencourt Uberti, Márcia Keske-Soares, Karina Carlesso Pagliarin

**Affiliations:** 1 Programa de Pós-graduação em Distúrbios da Comunicação Humana, Universidade Federal de Santa Maria – UFSM - Santa Maria (RS), Brasil.

**Keywords:** Speech, Child, Psychometrics, Speech Disorders, Digital Technology

## Abstract

**Purpose:**

To evaluate the psychometric properties of the naming and imitation tasks of the Digital Speech Assessment Instrument.

**Methods:**

Forty-five Brazilian Portuguese (BP)-speaking children aged 4 to 9 years, with and without Phonological Disorder (PD) participated in the study. Construct validity was verified by convergent validity, criterion validity was analyzed using Student's t-test for independent samples, and inter-rater reliability was assessed.

**Results:**

The naming task demonstrated satisfactory construct validity. In the imitation task, correlations ranged from moderate to nearly perfect. Both tasks discriminated well between groups, with children with PD presenting consistently lower scores. Inter-rater agreement was substantial to nearly perfect.

**Conclusion:**

The results indicate that the naming and imitation tasks of the Digital Speech Assessment Instrument are valid and reliable.

## INTRODUCTION

Speech is a complex process through which oral expression is made possible by the combination of linguistic, motor, and auditory characteristics^(1)^. Speaking is a dynamic motor skill that requires coordination between respiration, phonation, resonance, and articulation to achieve the production of words^(2)^. It involves rapid, systematic movements by articulatory organs such as the lips, jaw, tongue, and larynx. Speech develops over time as connections are established between motor and linguistic regions of the brain, resulting in the emergence of different aspects of processing and production^(3)^.

Speech is a complex process involving the interaction of cognitive, linguistic, motor, and auditory systems. At the cognitive level, speech production relies on brain areas for planning and organizing linguistic units. Linguistic processing selects and organizes words, while the motor component coordinates articulatory movements. The auditory component provides feedback to adjust these movements, ensuring speech accuracy. These interdependent processes are essential for effective speech production, with any impairment potentially leading to communication difficulties. Variations in these processes occur during development and can be disrupted by disorders, such as Speech Sound Disorders (SSDs), which include speech delays, errors, and Motor Speech Disorders (MSDs)^(4,5)^.

Speech delays refer to the delay in the acquisition and/or development of speech production processes (auditory and somatosensory representation). These conditions are predominantly of genetic origin and may be aggravated or attenuated by factors such as otitis media with effusion and/or developmental or psychosocial factors. Phonological Disorder (PD) falls into this category (speech delay) and is characterized by deficits in phonological knowledge^(5)^, manifesting as substitution and omission of consonants, along with consistency in speech errors, unlike Childhood Apraxia of Speech. In PD, there is an addition of sounds and distortion of sounds to facilitate production, transforming complex syllables into simpler ones^(6)^. PD is among the most common speech disorders in children^(7)^.

Speech errors are defined by articulation difficulties, as in the case of phonetic disorders. When speech delays and errors persist beyond the age of 9, they are referred to as Persistent Speech Delays or Persistent Speech Errors^(5,8)^. Motor Speech Disorders (MSDs) include Speech Motor Delay (SMD), Childhood Apraxia of Speech (CAS), and Childhood Dysarthria (CD) or the association of CAS and CD. Although these conditions present distinct features, they all involve motor disorganization in the process of transcoding (planning and/or programming) or motor execution. SMD and CD involve difficulties in the execution process, whereas CAS is associated with difficulties in the planning and programming of the motor patterns necessary for speech^(5)^. In contrast, SMD and CD are more related to execution issues. However, each condition manifests differently. The multiple factors involved in SSDs and our limited understanding of their causes or origins make the diagnostic process challenging^(6,9)^.

To ensure an accurate diagnosis, suspected SSD cases require a comprehensive evaluation approach that considers not only phonological aspects but also motor and cognitive factors. Such cases can only be evaluated by professionals with prior knowledge of typical speech acquisition and appropriate instruments to confirm this diagnostic hypothesis^(9)^. The tests used for this purpose must yield rapid, accurate, valid, and reliable results^(10)^. In Brazil, most instruments used for this purpose are paper-and-pencil based. These include the Child Phonological Assessment^(11)^ and the Child Language Test (ABFW)^(12)^. However, these tests require significant time for scoring and interpretation. To address such issues, studies have developed computerized assessment instruments (Phonological Assessment Tool (INFONO)^(13)^ and virtual tools (Phonological Assessment Tool (IAF)^(14)^ for native speakers of Brazilian Portuguese (BP) aged 9 years or younger. The psychometric properties of INFONO, including various types of validity and reliability, have already been established^(1,13)^. However, it is currently available only for research purposes.

The IAF is an online instrument, commercially available, and has recently undergone psychometric testing^(14-16)^. Although both tests are important and widely used in clinical and research settings, they focus exclusively on phonology, assessing only one aspect of SSDs. Additionally, they assess only children up to 9 years old. There is, therefore, a need for assessment instruments that explore older ages and other areas of speech production (word and pseudoword imitation and repetition) to analyze linguistic and motor variables. Such an approach could detect deficiencies in lexical retrieval and phonological encoding, as well as in the planning, programming, and/or execution of articulatory movements of speech, all of which may occur in SSDs. To obtain a comprehensive speech profile, it is essential to assess all levels of speech production^(17)^.

Given the need for assessment tools that explore older ages and other areas of speech production, the Digital Speech Assessment Tool was developed. The instrument was therefore developed to evaluate phonological and motor skills in children and adolescents who are native BP speakers, aged 2 to 17 years^(18)^.

Speech errors may follow different developmental trajectories. In young children, speech errors are often inherent to the developmental process and, in many cases, may persist beyond the expected age, thus characterizing SSD. In adolescents, some errors may persist for years without appropriate intervention. Furthermore, in certain cases, even with intervention, the prognosis may not be favorable, as seen in MSD, particularly in CAS, which continues to present speech distortions^(4)^.

The instrument includes tasks for naming, imitation, and repetition of words and pseudowords, providing a comprehensive approach to assess the linguistic and motor variables involved in speech production. It represents a significant advancement in the assessment of phonological and speech motor skills in Brazilian Portuguese-speaking children and adolescents. It is highly functional in clinical practice, requiring only a computer or tablet with internet access, which makes it accessible to a variety of professional settings. In doing so, it eliminates the need for paper-and-pencil assessments, contributing to more efficient service delivery. Moreover, it enables a comprehensive and accurate evaluation of speech abilities, including a wide range of real and pseudo-word stimuli, as well as the analysis of diadochokinesis. Additionally, this tool is currently undergoing standardization for adolescents, for whom no validated instrument is yet available.

The Digital Speech Assessment Tool has already undergone content validation and yielded satisfactory results^(18)^. However, it is still necessary to analyze other psychometric properties of the various tasks included in the instrument in order to establish it as a reliable assessment tool for clinical use. This study aims to evaluate the psychometric properties (validity and reliability) of the naming and imitation tasks of the Digital Speech Assessment Instrument.

## METHODS

This is a quantitative, cross-sectional, and correlational study, approved by the Research Ethics Committee of a higher education institution under protocol number 3.972.480, in accordance with Resolution 466/2012 of the National Health Council (CNS). Prior to the study, the parents/legal guardians of all potential participants were invited to read and sign an Informed Consent Form (ICF). Additionally, the children were only assessed after the Informed Assent Form was read and verbal consent was obtained.

### Participants

Participants were recruited for convenience and all attended public schools at the time of the study. Children with speech disorders were recruited from a university clinic, and children with typical development were selected through referrals from acquaintances.

Participants were selected based on the following inclusion criteria: speaking BP as the first language; age ranging from 2 to 9 years; appropriate neuropsychomotor development for their age; normal or corrected-to-normal visual acuity; normal auditory thresholds; adequate comprehension and expression of oral and/or written language for their age; no significant socioemotional disorders that could interfere with the study tasks; no oral myofunctional disorders that could interfere with speech production (e.g., lingual frenulum abnormalities); no diagnosed syndromes and/or genetic mutations; and no complex neurobehavioral disorders. Additionally, the clinical group was required to have a previous diagnosis of PD.

Socioemotional disorders were assessed through interviews with the guardians, considering disorders that might interfere with the study tasks. Neurobehavioral disorders were identified through self-reports by the guardians, who provided information about previous diagnoses or related behaviors. The participating children had already been referred from a department specializing in speech sound disorders, after undergoing an initial screening. During this screening, a clinical assessment was conducted, which led to a prior diagnosis of phonological disorder. This initial diagnosis served as a fundamental inclusion criterion for the children's participation in the study, ensuring that the sample group was homogeneous in relation to the condition being investigated. Thus, the subsequent assessment carried out within the scope of this study aimed to complement and confirm the understanding of these children's linguistic abilities, in line with the research objectives, rather than serving as an initial diagnostic step.

Participant selection was performed in the following steps: initial screening through a brief interview with a guardian to investigate the child's language and neuropsychomotor development, and to identify socioemotional disorders or neurobehavioral disorders. Instruments used included the Child Naming Test (TIN)^(19)^ to assess expressive language and long-term memory; the Auditory Vocabulary Test (TvAud33o)^(20)^ to assess receptive vocabulary; the Orofacial Myofunctional Assessment Protocol with Scores (AMIOFE)^(21)^ to examine the structure of the articulatory organs and stomatognathic functions; binaural auditory evoked potentials at 500, 1,000, 2,000, and 4,000 Hz, using an INTERACUSTICS AD229e audiometer; and INFONO to confirm the diagnosis of PD.

If children were outside the age ranges for TIN and TVAud, the researchers assessed expressive language and receptive vocabulary using clinical judgment during spontaneous speech. All children were evaluated with INFONO to confirm the PD diagnosis.

Data collection was performed by trained and qualified speech-language pathologists and undergraduate students.

The final sample consisted of 45 participants after the exclusion of six children: three for not attending to complete the assessments and three due to a previous diagnosis of a neurobehavioral disorder. The sample was divided into clinical and control groups. The clinical group comprised 26 children diagnosed with Phonological Disorder (PD), consisting of six females (23.1%) and 20 males (76.9%). The sample age ranged from four to nine years (M=6.12; SD=1.479). The age range of the sample was determined based on the studied PD. The control group consisted of 19 children without PD, including six (31.6%) females and 13 (68.4%) males. The age of this sample ranged from four to nine years (M=6.11; SD=1.663).

Criterion validity was assessed in a sample of 19 children with Typical Speech Development (TSD) (control group) and 26 children with PD (clinical group). To examine construct validity, 16 participants were recruited from the clinical group. The reliability analysis involved a sample of 20 children with PD from the clinical group.

### Instruments and procedures

Participants were subjected to the naming and imitation tasks of the Digital Speech Assessment Tool. Other tasks within this instrument will be investigated in future studies, as they were not fully developed and require additional adjustments.

The INFONO^(13)^ was used to confirm diagnoses and as the gold standard for convergent validity. INFONO is a speech assessment software program consisting of spontaneous naming, imitation, and chained speech tasks. The spontaneous naming and imitation tests each contain 84 target words, presented to the participant in the form of images. In the spontaneous naming task, the child is required to name the "animated" figure represented by gifs (a term used for animations composed of several compacted GIF images that give motion to the figure), illustrating the target word. Additionally, a key question is provided for the examiner to ask the child during the evaluation, such as: "What animal is this?" (for the word "horse"), and so on. In the imitation task, the child listens to the audio of the target word, which appears alongside a corresponding image.

The software allows the child's responses to be recorded in audio for transcription during or after the evaluation. Results are automatically generated (phonetic transcription, contrast analysis, severity of phonological disorder, and phonological process analysis).

The Digital Speech Assessment Tool is a website that can provide a more specific diagnosis of PD for speech-language pathologists. It contains tasks for figure naming (spontaneous speech elicitation using image naming), word and pseudoword imitation (immediate imitation of gestures and articulatory sounds), word and pseudoword repetition (five repetitions of previously recorded words and pseudowords), and diadochokinesis (production of monosyllabic, disyllabic, and trisyllabic sequences for five seconds, e.g., PATAKA). The Digital Speech Assessment Tool contains 91 real words in the naming tasks (61 words, including 4 monosyllabic words, 34 disyllabic words, 19 trisyllabic words, and 4 polysyllabic words), imitation (51 words, including 2 monosyllabic words, 26 disyllabic words, 20 trisyllabic words, and 3 polysyllabic words), and repetition (5 words). The criteria for selecting these words included acquisition order in BP^(1)^, ease of production, presence in the vocabulary of young children^(20)^, and representativeness^(18)^. The selected stimuli provide at least two opportunities for the production of each phoneme in BP in words that may vary in syllable count, stress patterns, and previous and subsequent contexts. The instrument also contains 26 pseudowords, created based on a study^(22)^, who developed and analyzed pseudowords in terms of present phonemes and production environment (favorable, neutral, or unfavorable). The author then used these words in the treatment of children with phonological disorders. The production of these words was assessed through imitation tasks (26 pseudowords) and repetition (four pseudowords). The data collected from the speech production tasks are recorded in audio during the evaluation for later analysis. This allows the examiner to access and transcribe the word or pseudoword afterward, as the instrument still does not perform automatic transcriptions and contrast analysis. The instrument underwent content validity testing and was developed through a seven-step process, consisting of: 1) Stimulus selection; 2) Expert judge analysis (24 speech-language pathologists with doctoral-level training and/or clinical experience in the field); 3) Semantic analysis; 4) Word illustrations; 5) Non-expert judge evaluation (children and adolescents); 6) Pilot study 1 (typically developing group); and 7) Pilot study 2 (groups with and without PD). The instrument showed satisfactory content validity^(21,23)^. Studies seeking validity and reliability evidence are crucial for determining the quality of a test^(10)^. Reliability represents the accuracy of the test through the reproducibility of a result consistently over time and space. Validity is understood as the ability of the instrument to accurately measure what is intended to be measured at each stage of the evaluation^(15)^. Currently, the naming and word repetition tasks are finalized and available on the test website, while the remaining tasks require additional adjustments.

In the naming task, for each word, the child is invited to name an image displayed on the computer screen. For each word in the naming task, the examiner asks the following question to the child: "What is this?" If the child does not produce the target word, a hint is provided to facilitate the identification and production of the target word. If the examiner still cannot elicit the word, they will produce the word and ask the child to repeat it (imitation). Examples include: "It’s a yellow fruit that monkeys like to eat"; "It’s an animal that makes honey and says bzzzzzz".

In the word imitation task, the stimuli are shown in a video focused on a mouth producing the target word, to avoid any distractions. The child is asked to watch and listen to the video and, once it is finished, is asked to say the word produced by the person in the video.

In both tasks, the examiner can immediately capture an audio recording of the participant's responses using the website itself, so that transcriptions can be made at the end of the test. Additionally, in the present study, the evaluations were recorded on video using QuickTime Player for later review of articulation and speech production for each patient. The administration duration for both tasks is approximately 20 minutes.

After the evaluation, transcriptions and contrast analysis for each child were performed manually, as the instrument still cannot generate automatic results. Transcription was performed after listening to the audio recording of the speech sample of all words that were saved at the time of the evaluation. The words were transcribed according to the International Phonetic Alphabet (IPA). Afterwards, the analysis was performed by calculating the Percentage of Correct Consonants - Revised (PCC-R). For the next stages of the project, the instrument will present ready-made transcription suggestions and the option to automatically transcribe the words.

### Data collection

Data collection and scoring were performed by two speech-language pathologists and three undergraduate students, all trained to apply the instruments. To administer the Digital Speech Assessment Tool, all examiners were trained in application, analysis, and scoring. The examiners completed 3 days of training where the instrument, application and analysis method were explained and everyone applied the instrument to themselves and to colleagues who did not participate in the project for testing. The entire training process was monitored and evaluated by the speech therapists responsible for the study. The other tests used in the research are covered in undergraduate courses, so all examiners had theoretical and practical knowledge of these instruments. A calibration assessment was carried out before the main study, with a random sample of 10 children.

### Data analysis

Construct validity was assessed using convergent validity. This refers to the verification that the assessment instruments used measure the same construct or concept. The justification for using convergent validity lies in the search for a substantial correlation between the results obtained by both instruments, ensuring that both are effective in measuring phonological productions, especially when analyzed in parallel. The transcribed responses were used to compile a database for the INFONO and the Digital Speech Assessment Tool, utilizing SPSS 30.0. The database included the total number of correct productions and the percentage of correct production for each phoneme in each position (Simple Onset, Coda, and Complex Onset). We also chose not to use the analysis of allophones in this study. The analysis of allophones, although an important tool in phonological investigation, was deliberately excluded from this study due to its geographical and sociolinguistic variability in the Brazilian context. In Brazil, allophones do not exhibit a homogeneous distribution across the country's regions. Therefore, the analysis of these phenomena might not reflect the reality of all children participating in the research, as the presence or absence of certain allophones may vary substantially depending on the sociolinguistic context.

The data were analyzed using the Pearson correlation coefficient to compare the percentage accuracy values for each phoneme between the Digital Speech Assessment Tool and INFONO^(13)^. The agreement rates were classified as follows: > 0.8 (almost perfect), 0.61–0.8 (substantial), 0.41–0.6 (moderate), 0.21–0.4 (fair), and < 0.2 (poor)^(24)^.

The criterion validity analysis involved a comparison between the control and clinical groups, matched by age, education, and gender. The results were analyzed using a non-parametric independent samples T-test.

Reliability between assessors was analyzed based on the first administration of the Digital Speech Assessment Tool by three experienced examiners trained to perform this evaluation and contrast analysis. These analyses were conducted independently. The evaluation recordings were provided to the examiners for transcription of speech samples and contrast analysis. The data were analyzed using the Kendall Tau correlation coefficient, which is appropriate for ordinal data, such as that obtained in transcriptions of speech productions. Significance was considered for p-values ≤ 0.05.

## RESULTS

Table 1 shows the correlations between the scores in the naming tasks of the Digital Speech Assessment Tool and INFONO. The correlations were significant and ranged from substantial to almost perfect (0.692 - 1.000) for phonemes in simple onset and coda positions. For complex onsets, the correlations ranged from moderate to almost perfect (0.430 - 1.000). The lowest correlation value was found for the cluster /kl/, while the highest values were observed for /t/ and /m/.

**Table 1:** Correlation between each phoneme in Naming tasks of the Digital Speech Assessment Instrument and the INFONO

**Phoneme**	**INFONO N**	**DSAI N**	**r**
**Simple onset**			
/p/	9	7	.
/b/	10	4	0.845^**^
/t/	19	10	1.000**
/d/	7	4	0.871**
/k/	18	11	0.993**
/g/	7	7	0.982**
/f/	7	5	0.795**
/v/	8	6	0.888**
/s/	9	7	0.928**
/z/	6	6	0.868**
/ʃ/	7	4	0.969**
/ʒ/	6	5	0.867**
/X/	6	5	0.982**
/m/	6	8	1.000**
/n/	8	5	.
/ɲ/	3	3	.
/l/	11	10	0.732**
/ʎ/	4	1	0.781**
/ɾ/- tap	8	8	0.853**
**Coda**			
/S/	10	5	0.692**
/ɾ/- tap	9	5	0.822**
**Complex Onset**			
/pr/	2	2	0.977**
/pl/	2	2	0.889**
/br/	2	2	0.961**
/bl/	1	1	0.550
/tr/	3	2	0.962**
/dr/	3	2	0.921**
/kr/	2	2	0.680**
/kl/	2	1	0.430
/gr/	3	1	0.830**
/fr/	3	2	0.675**
/fl/	2	1	0.913**
/vr/	1	1	1.000**

** ≤ 0.01;

. no variability

**Caption:** N = Total frequency of the phoneme in the INFONO and the Digital Speech Assessment Instrument (DSAI)

Table 2 shows the correlations between the word imitation tasks in the Digital Speech Assessment Tool and INFONO. The correlations were significant and ranged from moderate to almost perfect (0.428 - 1.000). The lowest correlation value was observed for /ʃ/ in coda position, while the highest values were found for the clusters /bl/ and /vr/.

**Table 2:** Correlation between each phoneme in the Imitation tasks of the Digital Speech Assessment Instrument and the INFONO

**Phoneme**	**INFONO N**	**DSAI N**	**r**
**Simple onset**			
/p/	9	6	.
/b/	10	4	0.978^**^
/t/	19	10	.
/d/	7	2	0.532^*^
/k/	18	7	0.989**
/g/	7	6	0.932**
/f/	7	4	0.965**
/v/	8	4	0.814**
/s/	9	6	0.801**
/z/	6	4	0.943**
/ʃ/	7	5	0.897**
/ʒ/	6	4	0.877**
/X/	6	2	0.972**
/m/	6	4	.
/n/	8	7	.
/ɲ/	3	2	.
/l/	11	9	0.837**
/ʎ/	4	2	0.523*
/ɾ/- tap	8	9	0.960**
**Coda**			
/S/	10	5	0.428
/ɾ/- tap	9	3	0.750**
**Complex Onset**			
/pr/	2	2	0.930**
/pl/	2	2	0.636**
/br/	2	2	0.914**
/bl/	1	1	1.000**
/tr/	3	2	0.963**
/dr/	3	2	0.912**
/kr/	2	3	0.590*
/kl/	2	1	0.767**
/gr/	3	2	0.876**
/fr/	3	2	0.743**
/fl/	2	1	0.636**
/vr/	1	1	1.000**

** ≤ 0.01;

* ≤ 0.05;

**.** no variability was observed

**Caption:** N = Total frequency of the phoneme in the INFONO and the Digital Speech Assessment Instrument (DSAI)

Table 3 shows the mean and standard deviation of the scores from the naming tasks in the control and clinical groups. The analysis revealed statistically significant differences between the groups (clinical and control) in speech production for all phonemes except /p/, /b/, /t/, /d/, /k/, /f/, /m/, /n/, and /ɲ/ in the initial onset position.

**Table 3:** Comparison between the performance of children with typical speech development and those with phonological disorders on the naming task of the Digital Speech Assessment Instrument

**Phoneme**	**Groups**		**p-value**
	**Clinical (N=26) Mean (SD)**	**Control (n=19) Mean (SD)**	
**Simple onset**			
/p/	98.01 (5.94)	100 (0.00)	0.100
/b/	96.15 (13.59)	100 (0.00)	0.161
/t/	98.46 (7.85)	100 (0.00)	0.399
/d/	96.46 (13.84)	100 (0.00)	0.204
/k/	89.09 (27.47)	100 (0.00)	0.054
/g/	85.68 (28.94)	100 (0.00)	0.018
/f/	89.23 (28.97)	100 (0.00)	0.070
/v/	86.22 (29.53)	100 (0.00)	0.025
/s/	82.55 (31.33)	99.25 (3.28)	0.012
/z/	79.23 (30.53)	100 (0.00)	0.002
/ʃ/	53.85 (45.66)	96.05 (17.21)	≤ 0.001
/ʒ/	59.62 (41.59)	94.74 (14.67)	≤ 0.001
/X/	82.12 (33.17)	100 (0.00)	0.011
/m/	99.52 (2.45)	100 (0.00)	0.399
/n/	95.07 (14.90)	100 (0.00)	0.104
/ ɲ /	97.42 (13.14)	100 (0.00)	0.399
/ʎ/	52 (50.99)	100 (0.00)	0.002
/ɾ/- tap	48.14 (40.08)	97.32 (6.78)	≤ 0.001
**Coda**			
/S/	74.01 (31.78)	100 (0.00)	≤ 0.001
/ɾ/- tap	49.94 (38.24)	98.95 (4.59)	≤ 0.001
**Complex onset**			
/pr/	32 (47.61)	92.11 (25.07)	≤ 0.001
/pl/	50 (44.72)	97.37 (11.47)	≤ 0.001
/br/	46.15 (48.83)	94.74 (15.77)	≤ 0.001
/bl/	47.06 (51.45)	100 (0.00)	≤ 0.001
/tr/	30.77 (42.61)	84.21 (37.46)	≤ 0.001
/dr/	21.15 (35.14)	68.42 (47.76)	≤ 0.001
/kr/	25 (40.62)	92.11 (18.73)	≤ 0.001
/kl/	28.57 (46.29)	100 (0.00)	≤ 0.001
/gr/	30.77 (47.07)	100 (0.00)	≤ 0.001
/fr/	28.85 (37.88)	94.74 (22.94)	≤ 0.001
/fl/	50 (51.08)	100 (0.00)	≤ 0.001
/vr/	23.08 (42.97)	100 (0.00)	≤ 0.001

Table 4 shows the mean and standard deviation of the scores for the control and clinical groups in the production of phonemes in simple onset, coda, and complex onset positions in the word imitation task. The analysis revealed statistically significant differences in speech production between the clinical and control groups for all phonemes except /p/, /b/, /t/, /d/, /k/, /g/, and /n/ in the simple onset position. The phonemes /m/ and /ɲ/ showed no variability, as all children in both groups produced them accurately.

**Table 4:** Comparison between the performance of children with typical speech development and those with phonological disorders on the word imitation task of the Digital Speech Assessment Instrument

**Phoneme imitation**	**Groups**		**p-value**
	**Clinical (N=26) Mean (SD)**	**Control (n=19) Mean (SD)**	
**Simple onset**			
/p/	98.71 (4.58)	100 (0.00)	0.161
/b/	95.19 (12.29)	100 (0.00)	0.057
/t/	99.19 (2.87)	100 (0.00)	0.162
/d/	98.08 (9.81)	100 (0.00)	0.399
/k/	92.64 (22.86)	100 (0.00)	0.113
/g/	90.36 (24.61)	100 (0.00)	0.057
/f/	93.85 (18.83)	98.68 (5.74)	0.226
/v/	81.73 (32.06)	96.05 (9.37)	0.039
/s/	83.59 (31.58)	100 (0.00)	0.014
/z/	88.19 (26.77)	100 (0.00)	0.034
/ʃ/	60.58 (46.74)	94.74 (18.67)	0.002
/ʒ/	65.19 (43.83)	93.42 (23.34)	0.008
/X/	82.69 (34.47)	100 (0.00)	0.017
/m/	-	-	-
/n/	98.08 (9.81)	100 (0.00)	0.399
/ɲ/	-	-	-
/l/	90.29 (14.69)	100 (0.00)	0.002
/ʎ/	69.23 (40.19)	92.11 (18.73)	0.015
/ɾ/- tap	50.17 (39.90)	98.83 (5.10)	≤ 0.001
**Coda**			
/S/	80.08 (30.19)	100 (0.00)	0.002
/ɾ/- tap	50.9 (39.82)	100 (0.00)	≤ 0.001
**Complex onset**			
/pr/	56.41 (47.63)	89.47 (26.77)	0.005
/pl/	46.15 (44.55)	100 (0.00)	≤ 0.001
/br/	40.38 (46.95)	89.47 (31.53)	≤ 0.001
/bl/	47.22 (49.92)	94.74 (22.94)	≤ 0.001
/tr/	40.38 (44.76)	86.84 (32.67)	≤ 0.001
/dr/	23.08 (38.03)	86.84 (32.67)	≤ 0.001
/kr/	31.36 (36.56)	91.23 (26.86)	≤ 0.001
/kl/	57.69 (50.38)	100 (0.00)	≤ 0.001
/gr/	42.31 (48.36)	92.11 (25.07)	≤ 0.001
/fr/	36.54 (45.95)	89.47 (31.53)	≤ 0.001
/fl/	50 (50.99)	100 (0.00)	≤ 0.001
/vr/	23.08 (42.97)	94.74 (22.94)	≤ 0.001

**-** not elicited due to lack of opportunities for production

Table 5 shows the inter-rater agreement for the naming task. The agreement rates ranged from substantial to almost perfect (63% to 100%). The phonemes with the lowest agreement rates were /d/, /b/, and /m/ in the simple initial onset position.

**Table 5:** Analysis of inter-rater agreement on the Naming task in the Digital Speech Assessment Instrument

**Phoneme**	**Kendall**	**p-value**
**Simple onset**		
/p/	1.00	≤0.001
/b/	0.66	0.003
/t/	1.00	≤0,001
/d/	0.63	0.004
/k/	0.86	≤0.001
/g/	1.00	≤0.001
/f/	0.82	≤0.001
/v/	0.71	≤0.001
/s/	0.88	≤0.001
/z/	0.89	≤0.001
/ʃ/	1.00	≤0.001
/ʒ/	0.80	≤0.001
/X/	0.88	≤0.001
/m/	0.69	0.003
/n/	0.81	≤0.001
/ɲ/	1.00	≤0.001
/l/	0.85	≤0.001
/ʎ/	1.00	≤0.001
/ɾ/- tap	0.88	≤0.001
**Coda**		
/S/	0.78	≤0.001
/ɾ/- tap	0.82	≤0.001
**Complex onset**		
/pr/	0.84	≤0.001
/pl/	0.87	≤0.001
/br/	0.95	≤0.001
/bl/	1.00	≤0.001
/tr/	0.94	≤0.001
/dr/	0.90	≤0.001
/kr/	0.78	≤0.001
/kl/	1.00	≤0.001
/gr/	1.00	≤0.001
/fr/	1.00	≤0.001
/fl/	1.00	≤0.001
/vr/	1.00	≤0.001

Table 6 presents the inter-rater analysis of each phoneme in the word imitation task. The agreement rates ranged from moderate to almost perfect (47% to 100%). The lowest rates were observed for /d/ in the simple onset position, where disagreements between examiners were more frequent.

**Table 6:** Analysis of inter-rater agreement on the word Imitation task of the Digital Speech Assessment Instrument

**Phoneme**	**Kendall**	**p-value**
**Simple onset**		
/p/	1.00	≤0.001
/b/	0.86	≤0.001
/t/	1.00	≤0.001
/d/	0.56	0.010
/k/	0.88	≤0.001
/g/	0.99	≤0.001
/f/	0.84	≤0.001
/v/	1.00	≤0.001
/s/	0.93	≤0.001
/z/	0.74	≤0.001
/ʃ/	0.83	≤0.001
/ʒ/	0.81	≤0.001
/X/	1.00	≤0.001
/m/	1.00	≤0.001
/n/	0.72	0.002
/ɲ/	1.00	≤0.001
/l/	0.70	≤0.001
/ʎ/	0.73	≤0.001
/ɾ/- tap	0.92	≤0.001
**Coda**		
/S/	0.92	≤0.001
/ɾ/- tap	0.92	≤0.001
**Complex onset**		
/pr/	0.92	≤0.001
/pl/	0.95	≤0.001
/br/	0.91	≤0.001
/bl/	1.00	0.001
/tr/	0.86	≤0.001
/dr/	0.88	≤0.001
/kr/	0.91	≤0.001
/kl/	0.82	≤0.001
/gr/	0.87	≤0.001
/fr/	0.94	≤0.001
/fl/	0.90	≤0.001
/vr/	0.89	≤0.001

## DISCUSSION

The Digital Speech Assessment Instrument was developed based on the Computer Articulation Instrument (CAI)^(17)^, a Dutch test that assesses speech production through various tasks (picture naming, imitation, word and pseudoword repetition, diadochokinesis), as well as two other phonological and motor speech assessment instruments^(13,25)^ developed in Brazil. The Digital Speech Assessment Instrument fills an existing gap in speech pathology by providing an assessment method for speech sound disorders (SSDs) in general, rather than just phonological disorders (PD)^(11,13)^. To date, only its content validity has been assessed^(21,22)^. Psychometric studies are essential to ensure that an instrument is reliable and valid^(10, 26)^. The present study aimed to collect evidence of construct validity, criterion validity, and inter-rater reliability for the word naming and imitation tasks of the Digital Speech Assessment Instrument, and positive results were obtained for all analyses. It is important to note that the remaining tasks in this instrument were not analyzed, as they were not finalized. Additionally, there are no validated digital/virtual instruments or software programs that assess pseudoword imitation, word and pseudoword repetition, or diadochokinesis, which could serve as a gold standard for comparison (convergent validity) in PD^(23)^. Most of the phonemes had substantial to almost perfect correlations, providing evidence of appropriate construct validity (Tables 1 and 2). Adequate correlations between the variables indicate that they capture what the instrument intends to measure^(26)^. The results regarding criterion validity (Tables 3 and 4) were statistically significant, as expected, demonstrating that both the word imitation and naming tasks were able to differentiate individuals with SSDs and typical speech (TS). Although the word imitation task did not reveal significant differences between the groups for plosive and nasal phonemes, this was expected since these phonemes had already been acquired by participants with TS. As plosives and nasals are early-acquired phonemes, it is expected that children with TS will not have significant difficulty in producing these sounds. These phonemes are among the first to emerge and solidify in the child’s phonological system. Plosives are the first phonemes acquired in Brazilian Portuguese and, like nasals, they are acquired before the age of three^(21)^. On the other hand, the later-acquired phonemes are those in which children with TS presented greater difficulty^(27)^. Thus, the results allow us to observe that the instrument is able to distinguish between children with and without PD. When analyzing the differences in performance of children in the word naming and imitation tasks, a significant improvement in the production of target words in the word imitation task can be highlighted. Children with TS benefit from visual and auditory cues in the task. A potential bias is evident in conducting this type of assessment task when administered in isolation, as a child’s production tends to improve when immediately prompted with the target stimulus^(28)^. Therefore, it should be used complementarily, combined with other types of tasks, to provide a more accurate diagnosis. This task also allows for assessing stimulability in children with SSDs. The stimulability measure is considered a complementary assessment to the SSD diagnosis and aims to detect whether a phoneme is present in the phonetic inventory that can be produced through imitation. Thus, a phoneme absent from the child’s phonological inventory but which can be produced through an imitative model is considered stimulable^(29)^. Inter-rater agreement was significant for all phonemes in the Digital Speech Assessment Instrument (Tables 5 and 6). The lowest agreement rates between raters were observed for the phoneme /d/ in the word imitation task, followed by /b/ in the naming task. As both are voiced plosives, this finding may indicate a perceptual difficulty on the part of the examiners exposed to these sounds. Discrimination between voiceless and voiced plosive phonemes is acoustically marked by the presence or absence of Voice Onset Time (VOT), along with other acoustic cues responsible for establishing the contrast in plosive voicing. This involves articulatory and acoustic refinement that may potentially hinder listener perception^(30)^. However, most of the tasks showed adequate reliability statistics, though the numbers were lower than those observed in INFONO^(18)^, whose inter-rater agreement rates (93% to 98%) suggested that the examiners were highly consistent in their ratings. The examination of the Digital Speech Assessment Instrument^(21,29)^ produced robust evidence of its construct validity, criterion validity, reliability, and inter-rater agreement. Therefore, the objectives of the present study were achieved, and an important contribution was made to the diagnosis of speech sound disorders by speech-language pathologists and researchers.

The present results should be interpreted in light of some limitations. The word imitation task is presented differently in the two instruments compared. In INFONO, the words are presented through audio recordings and animated images, while in the Digital Speech Assessment Instrument, a video of oral articulation is used without illustration of its meaning. This may have facilitated word production, especially for longer stimuli, as viewing articulatory movements can aid imitation. This hypothesis could be investigated in future studies. Additionally, there are no validated normative data for this task in INFONO, limiting the comparison of the obtained results. Another limitation of the study was the need for manual phonetic transcription by the raters, as access to the artificial intelligence technology required to automate this process was not yet available. We also emphasize that this study presented only part of the validation process of the instrument, which must be completed to ensure its full psychometric robustness. Finally, this instrument should be applied to other criterion groups, including children with motor speech delays, childhood apraxia of speech, and dysarthria, i.e., other types of SSDs. It should also be tested in other age ranges, as the instrument evaluates up to adolescence, and this study only assessed children. However, such studies can only be conducted once the instrument is finalized.

It is important to note that the phonemes /n/ and /l/ in coda position were transcribed but not analyzed, as the contrastive analysis did not take these codas into account. This is a limitation of our study, as it may have influenced the accuracy of our counts. The authors are aware of the need to evaluate these phonemes in other SSDs. However, the present study only included children with SSDs, who rarely make omission or substitution errors in the pronunciation of these phonemes.

This instrument has significant clinical implications for speech-language pathology practice. The digitization of the test can reduce the time required for administration and interpretation of results, enabling professionals to conduct a greater number of assessments in a shorter period without compromising quality. The digital nature of the instrument ensures that all evaluators follow a standardized protocol, thus increasing consistency across assessments and providing rapid results, which facilitates immediate feedback for children and their caregivers.

Moreover, the instrument allows for regular assessments over time, making it easier to monitor children’s progress and the effectiveness of interventions. With precise data on speech performance, therapists can more effectively tailor interventions to meet the individual needs of each child. The collected data can also be used to develop evidence-based assessment and treatment protocols, contributing to the establishment of more robust clinical practices.

Additionally, this information can serve as a foundation for research that deepens the understanding of speech disorders, promoting advances in the field. The incorporation of the instrument into training programs for speech-language pathologists increases professionals’ familiarity with digital technologies, enriching their clinical practice. Thus, a digital speech assessment instrument not only modernizes the clinical approach but also improves the effectiveness of interventions, promoting child-centered care that addresses their specific needs.

Although there are limitations that need to be addressed in future studies, such as the need for validation of normative data and the analysis of additional phonemes, the clinical implications of the instrument are significant and offer considerable potential for improving care for children with Speech Sound Disorders (SSD).

## CONCLUSION

The naming and imitation tasks of the Digital Speech Assessment Instrument demonstrated satisfactory validity, as evidenced by their correlation with another established instrument used in the assessment of SSD. Inter-rater reliability was also found to be adequate, which is essential to ensure consistency across assessments and improve diagnostic accuracy.

Nonetheless, further research is necessary to examine additional psychometric properties of the instrument, such as test-retest reliability, to assess the validity and reliability of the remaining tasks, and to confirm its applicability across more diverse clinical and population settings.

This study represents an important step toward the validation of the Digital Speech Assessment Instrument, offering a promising tool for speech-language pathologists in the diagnosis and monitoring of SSD, with the potential to enhance clinical decision-making and service delivery.
